# Learning Morse Code Alters Microstructural Properties in the Inferior Longitudinal Fasciculus: A DTI Study

**DOI:** 10.3389/fnhum.2017.00383

**Published:** 2017-07-26

**Authors:** Lara Schlaffke, Alexander Leemans, Lauren M. Schweizer, Sebastian Ocklenburg, Tobias Schmidt-Wilcke

**Affiliations:** ^1^Department of Neurology, BG-Kliniken Bergmannsheil, Ruhr Universität Bochum Bochum, Germany; ^2^Department of Radiology, University Medical Center Utrecht Utrecht, Netherlands; ^3^Image Sciences Institute, University Medical Center Utrecht Utrecht, Netherlands; ^4^Department of Biopsychology, Ruhr Universität Bochum Bochum, Germany; ^5^Department of Neurology, St. Mauritius Therapieklinik Meerbusch, Germany; ^6^Institute of Clinical Neuroscience and Medical Psychology, Medical Faculty, University of Düsseldorf Düsseldorf, Germany

**Keywords:** DTI, semantic learning, inferior longitudinal fasciculus, Morse code, language learning

## Abstract

Learning relies on neuroplasticity, which has mainly been studied in gray matter (GM). However, there is mounting evidence indicating a critical role of white matter changes involved in learning processes. One of the most important learning processes in human development is language acquisition. However, due to the length of this learning process, it has been notoriously difficult to investigate the underlying neuroplastic changes. Here, we report a novel learning paradigm to assess the role of white matter plasticity for language acquisition. By acoustically presenting Morse Code (MC) using an in house developed audio book as a model for language-type learning, we generated a well-controlled learning environment that allows for the detection of subtle white matter changes related to language type learning in a much shorter time frame than usual language acquisition. In total 12 letters of the MC alphabet were learned within six learning session, which allowed study participants to perform a word recognition MC decoding task. In this study, we found that learning MC was associated with significant microstructural changes in the left inferior longitudinal fasciculus (ILF). The fractional anisotropy (FA) of this associative fiber bundle connecting the occipital and posterior temporal cortex with the temporal pole as well as the hippocampus and amygdala was increased. Furthermore, white matter plasticity was associated with task performance of MC decoding, indicating that the structural changes were related to learning efficiency. In conclusion, our findings demonstrate an important role of white matter neuroplasticity for acquiring a new language skill.

## Introduction

Learning relies on neural plasticity, which is thought to rely mainly on gray matter (GM) changes. Among these, changes in GM density have been reported for second language learning (Li et al., 2014). Furthermore, differences in the hippocampus are shown to be related to the navigation ability of London taxi drivers (Maguire et al., 2000) as well as to extensive exercise in martial arts (Schlaffke et al., 2014). Modification of GM volume is also known to occur due to cognitive learning (Draganski et al., 2006; Ceccarelli et al., 2009) and motor skill learning (Draganski et al., 2004; Gryga et al., 2012). The cytoarchitectonic underpinnings of these observed changes in either GM density or volume, however, have still not been completely elucidated. Astrocytic swelling, growing synapses, increased blood flow as well as neurogenesis have been discussed (Johansen-Berg et al., 2012).

It is clear that complex learning processes cannot be completely understood by looking at structural changes in isolated GM regions. Also the connections between these areas need to be taken into account as there is increasing evidence that not only GM plasticity but also changes in white matter are important in the context of learning processes (Taubert et al., 2010; Jolles et al., 2016). White matter can be investigated and quantified using diffusion tensor imaging, which provides indirect information of these structures by analyzing water diffusion along axons. However, white matter plasticity can also rely on different cytoarchitectonic changes, which underlie different temporal dynamics. Modification of synapses and their associated dendritic spines would only take seconds to minutes (Johansen-Berg et al., 2012) and would lead to different outcome parameters when analyzing diffusion in white matter structures. In a study by Sagi et al. (2012), it was demonstrated that even 2 h of practicing a spatial learning task leads to a decrease in mean diffusivity (MD) in the hippocampus and parahippocampal gyrus. A study by Bengtsson et al. (2005) showed that for piano players, age has an impact on correlations between the fractional anisotropy (FA) and performance.

One of the most important learning processes in human development is language learning. Language learning and processing is known to recruit a vast network of mostly left hemispheric brain regions. These include the inferior frontal gyrus, especially Broca’s area (Broca, 1861), as well as posterior superior temporal regions, known as Wernicke’s Area (Wernicke, 1874). Based on lesion studies, both areas are known to be critically involved in speech production and speech processing. However, there is also emerging evidence, that the fusiform gyrus is also involved in language processing due to its role in letter conversion (Friederici, 2011) and letter categorization (Pernet et al., 2005). Although brain imaging methods have enabled the investigation of specific brain areas and their role in language generation and comprehension, due to the length of this learning process, it has been notoriously difficult to investigate associated neuroplastic changes in white matter.

A crucial prerequisite for these anatomically separated language areas to work together as a network are their interconnections via the white matter pathways. Several different tracts have been shown to play important roles in the language network and therefore are also likely to be involved in language acquisition. The arcuate fasciculus (AF) was initially described with its role in language networks in 1874 by Wernicke as it was initially thought to connect Broca’s to Wernicke’s area (Wernicke, 1874), but is now known to be a large and highly complex multi segment pathway connecting several associative areas (Catani and Mesulam, 2008; Bernal and Ardila, 2009). Following research by Lichtheim (1885) and Geschwind (1970), the important role of this associative fiber bundle is the connection of auditory and motor word comprehension regions (Lichtheim, 1885; Graves, 1997). In addition to the AF, it was demonstrated that the inferior longitudinal fasciculus (ILF), the fasciculus uncinatus (UNC) as well as the inferior fronto-occipital fasciculus (IFOF), mediate the connection between semantic processing and language networks (Saur et al., 2008).

The ILF is an associative fiber bundle, which connects the lateral occipital lobe with the temporal lobe. Due to its termination in the visual-word-form area the ILF is highly involved in face recognition as well as in reading processes like letter recognition. It is also thought to play a role in linking object representations to their lexical labels (Mummery et al., 1999). The UNC is also an associative bundle which interconnects the anterior temporal lobe to the orbitofrontal area, including the inferior frontal gyrus (Catani et al., 2002), and is thought to be involved in emotion processing. The UNC may play an important role in lexical retrieval and semantic associations, and aspects of naming that require connections from temporal to frontal components of the language network (e.g., the naming of actions; Lu et al., 2002; Grossman et al., 2004). The IFOF is the only direct connection between occipital and frontal cortex in the human brain (Catani et al., 2007) and likely to be involved in reading and attention processing (Catani and Thiebaut de Schotten, 2008).

The aim of the current study was to investigate language-learning related changes in white matter structures. Normal acquisition of a first or second language is a complex multi-year process that is highly individual and strongly depends on individual experience, age and previous knowledge (Schlegel et al., 2012). Thus, this learning process is highly difficult to investigate in neuroimaging studies. In this study we used a Morse Code (MC) paradigm as a model for language-type learning. It was recently shown, that MC is useful to induce functional neuroplastic adaptation in regions relevant for reading and language comprehension and language learning (Schlaffke et al., 2015). By using MC, we generated a well-controlled learning environment that allows for determining white matter changes related to language learning in a much shorter time frame than usual language acquisition. MC is an international information transmitting system in which letters of the Roman alphabet are represented as sequences of long or short signals. Thus, linguistic information of any complexity level can be presented to the skilled recipient using MC, making it an ideal tool to investigate neuroplastic changes associated with language type learning. Since all participants started without any knowledge of MC, this paradigm is well controlled for possible effects of previous experience. This allows the interpretation of post-learning performance in a MC-decoding task as a measure of newly gained knowledge. In our previous study, we could show, that learning MC leads to functional plasticity in language related brain regions (i.e., Broca’s area and fusiform gyrus, Schlaffke et al., 2015). From a network perspective, however, we assume that also changes in white matter occur due to learning MC. Therefore, in this study, using diffusion tensor imaging, our goal was to quantify white matter plasticity with respect to its role in language type learning and identify learning related white matter association. More specifically, the FA of the ILF was investigated to provide evidence for dynamic changes in language-type learning.

## Materials and Methods

### Study Participants

In this study, 16 healthy, right handed participants (23 ± 2 years; 10 males) with inconspicuous T1-weighted images underwent diffusion weighted imaging both before and after learning 12 letters of acoustically presented MC. Informed written consent was obtained from all study participants prior to study enrollment. The study was approved by the ethics committee of the faculty for psychology of the Ruhr-University Bochum, Germany; Number: 061 from April 11th, 2013.

### Learning and Task

To teach the participants MC, we used an in house developed audiobook, containing six sessions (*ca*. 1 h per session) to learn 12 letters of MC (day 1: E S N and O; day 2: T and R; day 3: U and D; day 4: A and I; day 5: M and G; day 6: repetition of all letters, see Schlaffke et al., 2015 for detailed description of the learning process and task). The learning sessions were conducted in six consecutive days, with an interception of maximal one weekend, resulting in a learning period of maximal 8 days (e.g., Mon–Fri Session 1–5 followed by session six on the next Monday, or Wed–Fri session 1–3, followed by session 4–6 on Mon–Wed). After the learning sessions all participants were able to decode words consisting of three of these 12 letters. During the task, auditory presented three-letter trains of MC had to be translated and decoded to decide whether they made up a word or not via button press in the MR scanner. The three-letter trains were presented jittered within blocks of nine sand randomized for each session and subject. The percentage of correct answers in this task was defined as performance.

### Image Acquisition

Magnetic resonance imaging (MRI) was performed on a 3.0 Tesla MR scanner (Philips Achieva 3.2, Best, Netherlands) using a 32-channel head coil, before the first learning session (the same day) and at the day following the last learning session. Diffusion weighted imaging was performed using 32 diffusion weighted gradient directions (*b* = 1000 s/mm^2^) with one non-diffusion weighted image. For all gradient directions two volumes were acquired and averaged for a better SNR yielding a total acquisition time of approximately 10 min (FOV = 224 × 224 × 120 mm^3^, 2 × 2 × 2 mm^3^ voxel size, yielding 60 slices, TR 8151 ms, TE 88 ms). In addition, a high resolution (1 × 1 × 1 mm^3^ voxel size) T1-weighted image was acquired as a reference for EPI-distortion correction (TR = 8.3 ms, TE = 3.8 ms Flip angle 9°, FOV 256 × 256 × 220 mm^3^).

### DTI Data Processing

All processing steps were conducted using the *ExploreDTI* toolbox (Leemans et al., 2009). After correcting for subject motion, distortions due to eddy currents and EPI deformations, REKINDLE (Tax et al., 2015) was used to detect and remove outliers in combination with the weighted linear least squares estimation approach (Veraart et al., 2013) to compute the diffusion tensor (Leemans and Jones, 2009; Irfanoglu et al., 2012). To correct for EPI distortions, the diffusion-weighted images were non-rigidly aligned (image contrast during registration is the FA) to the subjects’ individual high resolution T1-weighted image, with the deformation field constrained along the phase encoded A-P axis.

### Whole Brain Tractography

Whole-brain fiber tractography was performed as described by Basser et al. (2000). A 2 × 2 × 2 mm^3^ seed resolution grid was used with minimum FA value of 0.2, angle threshold of 30°, and step size of 1 mm.

### Automated Fiber Bundle Segmentation

For a non-biased extraction of the fibers of interest (ILF, IFOF and UNC) one representative subject (template) was chosen for an initial creation of AND-ROIs, meaning that only fibers, which pass all of these ROIs were extracted. For the extraction of the ILF an initial ROI was placed on a coronal slice in the anterior horn of the temporal lobe, a second ROI in a coronal slice of the posterior temporal lobe at the junction of Brodmann areas 20/21/37. The IFOF was extracted by placing one ROI in a coronal slice of the inferior frontal lobe and another one on a coronal slice of the posterior medial temporal lobe. To extract the UNC two ROIs were placed in the same coronal slice in the anterior temporal lobe and the frontal lobe (as recommended in the *ExploreDTI* manual). For all fiber extractions, an additional NOT-ROI was set in the medial longitudinal fissure, to exclude fibers, which pass this fissure. Using the atlas based tractography segmentation plug-in, implemented in *ExploreDTI*, these ROIs were than warped onto each individual’s space by applying the transformation matrix generated by a realignment of the template FA map to each individuals’ FA map in native-space. We used the whole tracts instead of segments between the ROIs and performed visual inspection of tracking quality, e.g., no tracts missing, no additional tracts found, to ensure comparable tract information. For the extracted tracts, the mean FA, MD, approximate tract volume and the number of streamlines were computed and statistically compared for the two measurements pre and post learning as well as for the tracts in the left and right hemisphere by applying a student’s paired *t*-test.

### Asymmetries

The laterality index (LI) of the FA for the tracts of interest was calculated using the following equation: $LI=\frac{FA_{\text{left}}-FA_{\text{right}}}{FA_{\text{left}}+FA_{\text{right}}}$.

## Results

### Performance

The learning intervention consisting of six learning sessions successfully allowed the study participants to decode the learnt 12 letters of MC as indicated by the low number of errors made in the last learning session (<10%). Furthermore, the three-letter decoding task during an fMRI scan showed an increase in performance from 42.6% (SEM ± 1.45, of 50% guessing chance prior to learning) to 76.9% (SEM ± 2.80, *p* < 0.001) post learning (see Schlaffke et al., 2015 for details).

### Tractography

**Figure 1:**
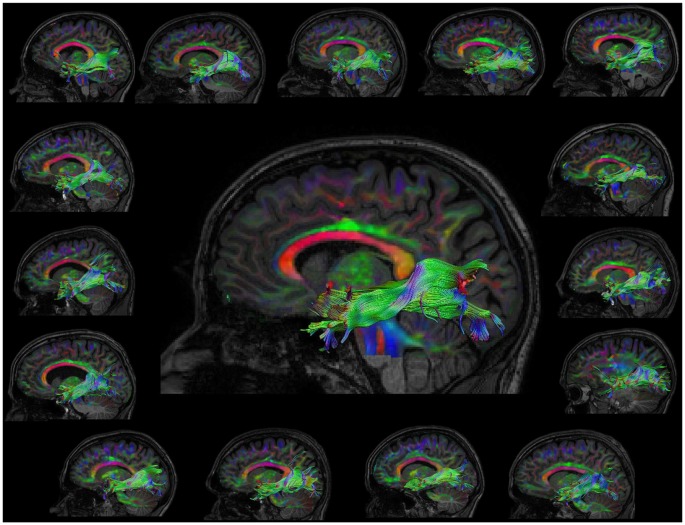
Visualization of the left inferior longitudinal fasciculus (ILF) post learning for all subjects. The representative subject, which was initially used to define ROIs is shown in the middle.

The automated atlas based tractography segmentation correctly identified the tracts of interest (ILF, UNC and IFOF; left and right; pre and post) for all subjects. Figure 1 shows the correctly identified ILF of all subjects.

Properties of the analyzed tracts (number of fibers, volume, FA, MD) were statistically compared pre and post learning as well as between left and right hemisphere using student’s paired *t*-tests. Correlation analysis (Pearson) was performed to test for significant correlation coefficients between tract properties and MC performance.

### Longitudinal Changes in White Matter Structure

**Figure 2:**
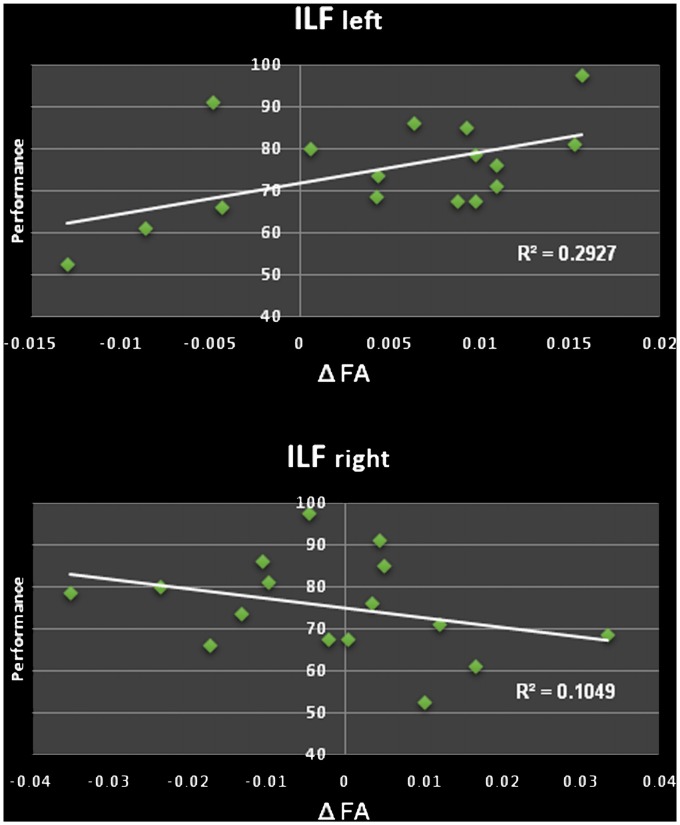
Top: positive correlation of changes in the fractional anisotropy (FA; Δ FA) in the left ILF and performance in the word/non-word discrimination task. Bottom: non-significant correlation of FA changes FA (Δ FA) in the right ILF and performance of the word/non-word discrimination task.

We found a significant increase of the mean FA (from 0.487 to 0.492, *p* = 0.022) of the left ILF when comparing the ILF pre and post the learning period, going along with an increase of the first eigenvalue (*λ*_1_ from 1.229 to 1.236 × 10^−3^ mm^2^/s, *p* = 0.044). This was neither seen in the right ILF (*p* = 0.322) nor in the IFOF or UNC (bilateral). Furthermore, this increase correlated significantly with the performance e.g., the better study participants performed in the word/non-word discrimination task after learning the more the FA in the left ILF increased (Δ FA), the (*r* = 0.541, *p* = 0.015; see Figure 2). There were no significant changes in the number of streamlines, tract volume or tract length.

### Asymmetry Results

Since the association between changes in FA and performance was only observed in the left ILF, we subsequently focused on laterality differences and compared left and right tract properties for both pre and post learning data. The data of each tract are shown in the Table 1.

**Table 1:** Quantitative parameters of the analyzed fiber tracts.

	Pre (left/right)			Post (left/right)		
Fiber Bundle	ILF	UNC	IFOF	ILF	UNC	IFOF
Mean FA	0.487/0.474	0.444/0.458	0.535/0.543	0.492/0.472	0.447/0.458	0.533/0.535
Mean MD (*10^−3^ mm^2^/s)	0.772/0.769	0.752/0.769	0.758/0.768	0.774/0.767	0.750/0.771	0.758/0.764
approximate tract volume mm^3^	9125/7418	1984/1973	6397/8402	9359/7289	1990/2062	6751/8432
Number of fibers	565.63/427.25	94.44/111.69	307.50/491.25	570.13/413.00	94.93/113.44	334.81/506.31
Mean tract length (mm)	98.72/103.41	79.02/76.07	155.63/148.87	97.53/101.35	82.25/77.10	155.72/149.67

*ILF, inferior longitudinal fasciculus; UNC, Fasciculus uncinatus; IFOF, inferior fronto-occipital fasciculus*.

The IFOF as well as the UNC showed significantly higher MD values in right compared to left tracts. This holds true for both time points. The IFOF additionally showed a significantly higher tract volume in the right hemisphere as compared to the left. This is in line with results reported earlier by Thiebaut de Schotten et al. (2011) and will not be discussed further.

**Figure 3:**
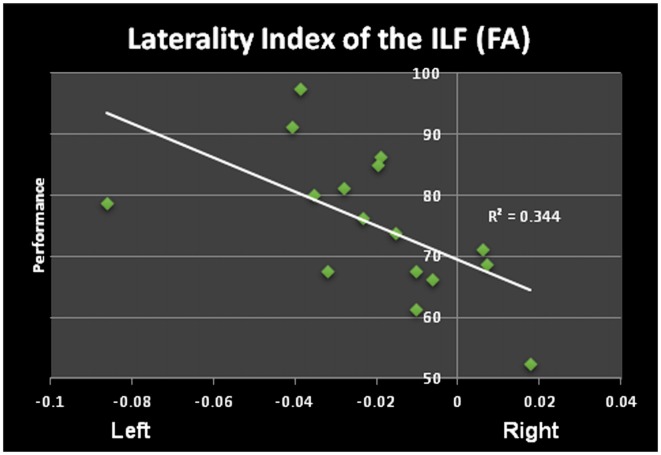
Significant correlation of the laterality index (LI) of the FA of the ILF and performance in the word/non-word discrimination task.

The ILF shows a significant leftward asymmetry observed by a significantly higher FA as well as number of streamlines and tract volumes (no differences in tract length) pre and post learning. Furthermore the differences between left and right FA ($LI=\frac{FA_{\text{left}}-FA_{\text{right}}}{FA_{\text{left}}+FA_{\text{right}}}$) post learning are significantly correlated with performance, e.g., the higher the FA in the left ILF as compared to the right ILF, the better study participants perform the word/non-word discrimination task (*r* = −0.5865, *p* = 0.008; see Figure 3). This correlation was not found, when comparing the LI pre learning with the task performance. The LI of the FA of the ILF showed a shift towards left (LI from −0.014 to −0.021), which however was not found to be significant (*p* > 0.1).

## Discussion

In this study we used MC learning as a novel paradigm to investigate white matter plasticity in language-related white matter pathways for language-type learning. We found significant relationships between learning efficiency, measured by performance in a word/non-word discrimination task and increase in FA in the left ILF. Thus, our data implicate a role of white matter plasticity for learning new language-type skills, a finding that is well in line with DTI studies investigating other forms of learning. For example, a 2 month period of math learning led to plastic changes in the perisylvian network (Jolles et al., 2016). A relation of skill learning and plastic changes in white matter microstructure was further supported by a DTI study by Scholz et al. (2009) who reported that learning how to juggle led to an FA increase in the WM located underneath the intraparietal sulcus.

Changes in white matter, observed by changes in FA or MD, were not only reported for long term paradigms, e.g., weeks or months, but also for very fast learning paradigms, e.g., hours. Sagi et al. (2012) demonstrated that a 2-h spatial learning task already leads to gender-specific decrease of MD in hippocampal regions. Together these findings suggest that changes in white matter may underlie different neurobiological mechanisms and dynamics (Sagi et al., 2012).

An important aspect of the present results is the fact that learning MC did not only lead to plastic changes in the ILF, but that these changes were also related to performance. In addition, no significant changes or relationships were found for the UNC or the IFOF, allowing the assumption that these changes in the ILF were related to the learning process.

A cross sectional study investigating the association between structural properties and behavioral measures using tract based statistics showed, that math-scores correlate with white matter properties in the parietal cortex in grade 10 and 11 adolescents (Matejko et al., 2013). Moreover, Bengtsson et al. (2005) could show that extensive piano practice leads to differential white matter properties in childhood, adolescence and adulthood. Importantly, the authors also found a positive relation between the amount of practice and white matter microstructure in different tracts, depending on age. While the correlation between behavioral measures and white matter properties found in our study is in line with the piano study by Bengtsson et al. (2005), our study is the first to report such a finding for language-type learning within a longitudinal study design.

One striking aspect of this structure-performance relationship is the finding that performance was also correlated with the lateralization index of the ILF post learning, i.e., the better study participants performed in the task, the more left lateralized the FA in the ILF was. None of these correlations was found in the UNC or the IFOF, further associative fiber bundles, which are expected to play a critical role in lexical and semantic processing. This link between asymmetry and performance is generally in line with previous research implicating different functional roles for the left and the right ILF.

The left ILF is known for its critical role in language processing as well as in reading (Catani and Mesulam, 2008). Degeneration of the ILF was shown to lead to word blindness (Epelbaum et al., 2008) and to deficits in lexical retrieval tasks in progressive aphasia (Powers et al., 2013). In contrast, the right ILF is thought to play a major role in verbal memory rather than language or semantic processing (Shinoura et al., 2011). In general, the left and the right hemisphere have differential functional and structural network properties, as shown by both graph theoretical analysis (Iturria-Medina et al., 2011; Caeyenberghs and Leemans, 2014) and resting state connectivity analysis (Gotts et al., 2013).

On the level of individual tracts, structural asymmetries in the AF have been shown to be relevant for performance in language based tasks, such as verbal recall (Catani et al., 2007), phonological processing and the *Peabody Picture Vocabulary Task* (Lebel and Beaulieu, 2009). Moreover, AF asymmetries have been shown to be related to functional language lateralization (Powell et al., 2006; Vernooij et al., 2007; Propper et al., 2010; Ocklenburg et al., 2013, 2014; Sreedharan et al., 2015).

Similarly, structural asymmetries in the UNC have not only been shown to be functionally relevant in language processing (Ocklenburg et al., 2013, 2014), but also in relation to stress responses (Madsen et al., 2012). For the ILF, in a developmental study, Song et al. (2015) reported a leftward FA asymmetry in this tract starting 40 weeks after gestation (Song et al., 2015). Regarding the functional significance of the asymmetry in the ILF, James et al. (2015) could show a positive correlation with functional language lateralization, implicating a functional relevance of ILF asymmetries for the language systems. In a study by Xiang et al. (2015), it was not only shown that learning a second language leads to a shift in laterality, which correlates with the second language proficiency, but also that changes in the perisylvian networks occur after a learning period of 6 weeks, suggesting fast dynamic changes.

Investigating a large sample of more than 1800 participants, Hirnstein et al. (2014) could show that subjects with stronger functional lateralization in the dichotic listening task, also had higher overall accuracy, implicating more efficient language processing in strongly lateralized subjects. Our data show, that this also seems to be the case for structural asymmetries in the ILF, a language relevant white matter pathway.

Our finding that stronger structural asymmetries in the ILF were related to better performance in the word/non-word discrimination task is also in line with studies investigating the relation of hemispheric asymmetries to behavioral performance. In general, hemispheric asymmetries are thought to be beneficial to overall brain efficiency by avoiding processing conflicts between the two hemispheres (Levy, 1969) increasing processing speed (Ringo et al., 1994) as well as enhancing parallel processing (Rogers et al., 2004). However, the underlying mechanism still needs to be fully elucidated.

Diffusion MRI allows the investigation of white matter structures, since estimated diffusion metrics in each voxel are highly dependent on fiber microstructure. However, various factors, such as myelination, fiber thickness, -length and -density would affect the diffusion profile, therefore an explicit discrimination of the underlying changes cannot satisfactorily be ensured. Multiple mechanisms underlying various temporal dynamics like astrocytic swelling, synaptic plasticity as well as myelination are possible.

Observed DTI changes in rats after spatial navigation training give evidence for the structural plasticity that occurs in astrocytes and the potential of MRI for probing structural neuroplasticity and hence indirectly localizing LTP (Blumenfeld-Katzir et al., 2011). Aside from this, there is not much evidence, so far, for interpreting the neural correlates of observed diffusion changes and further comparative research with animal models need to be done.

In a previous study we could show that MC learning leads also to functional changes when processing acoustically presented MC-stimuli, by allowing a higher cognitive (and language related) network to be activated (Schlaffke et al., 2015). We now extent these findings by showing that white matter pathways that connect regions within this network show learning related changes in terms of white matter plasticity.

In summary, our data show learning dependent changes in white matter in language related fiber tracts. This demonstrates that longitudinal DTI analyses may provide new insights in neurobiological underpinnings that cause changes observed in functional MRI investigations.

## Conclusion

Our data provide strong evidence that MC learning leads to changes in white matter structure. The observed leftward asymmetry and relation to performance indicates that MC learning represents a form of language learning. Importantly, MC learning is a much better controlled and also much faster paradigm to experimentally assess language-type acquisition then for example following natural learning of a second language. In a previous study we could show that MC learning leads also to functional changes when processing acoustically presented MC-stimuli, by allowing a higher cognitive (and language related) network to be activated (Schlaffke et al., 2015). We now extent these findings by showing that white matter pathways that connect regions within this network show learning related changes in terms of white matter plasticity.

## Author Contributions

LS and TS-W developed study conception and design. TS-W: principal investigator. LS, LMS, SO and AL: interpretation of data, wrote the manuscript. LS and LMS acquired data. LS, AL and SO performed data analyses. LS, SO and LMS performed statistics. TS-W and AL made critical revisions. All authors contributed to and have approved the final manuscript.

## Conflict of Interest Statement

The authors declare that the research was conducted in the absence of any commercial or financial relationships that could be construed as a potential conflict of interest.
